# Supplementary material for: Accurate prediction of X-ray pulse properties from a free-electron laser using machine learning
Source: Nat Commun. 2017 Jun 5;8:15461. doi: 10.1038/ncomms15461 (PMC5465316; doi:10.1038/ncomms15461)
Supplement: Supplementary Information — Supplementary Notes, Supplementary Table, Supplementary Figures and Supplementary References [file ncomms15461-s1.pdf]

**Supplementary Note 1. Machine learning in the context of this paper.**

Machine learning[1] as applied in this paper falls under the category of supervised learning for regression. Given a vector of $n_\mathrm{f}$ input variables $\mathbf{x} = (x_1, x_2, ..., x_{n_\mathrm{f}})$, known as features, and a vector of $n_\mathrm{t}$ output variables $\mathbf{y} = (y_1, y_2, ..., y_{n_\mathrm{t}})$, known as targets, it attempts to find a model $f$ that relates the features and the targets as $\mathbf{y} = f(\mathbf{x})$. This can be defined as the following optimization problem:

$$f = \arg\min_{f^*} E[\|\mathbf{y} - f^*(\mathbf{x})\|^2] \tag{1}$$

where $E$ indicates the expected value with respect to the distribution of the data. Since a generic function approximator $f_\mathbf{a}$ defined in terms of a series of parameters $\mathbf{a}$ is normally used, the problem reduces to finding the parameters $\mathbf{a}$ of the function that solve the optimization problem:

$$\mathbf{a} = \arg\min_{\mathbf{a}^*} E[\|\mathbf{y} - f_{\mathbf{a}^*}(\mathbf{x})\|^2] \tag{2}$$

In supervised learning for regression the parameters of the model are optimized based on some training data, consisting of a series of $n_\mathrm{e}$ training examples with associated values for the features $\{\mathbf{x}^1, \mathbf{x}^2, ..., \mathbf{x}^{n_\mathrm{e}}\}$ and their corresponding target values $\{\mathbf{y}^1, \mathbf{y}^2, ..., \mathbf{y}^{n_\mathrm{e}}\}$. This allows approximately redefining the previous optimization problem in terms of the training data as:

$$\mathbf{a} = \arg\min_{\mathbf{a}^*} \frac{1}{2n_\mathrm{e}} \sum_{i=1}^{n_\mathrm{e}} \|\mathbf{y}^i - f_{\mathbf{a}^*}(\mathbf{x}^i)\|^2 = \arg\min_{\mathbf{a}^*} J(\mathbf{a}^*) \tag{3}$$

where $J(\mathbf{a})$ is known as the cost function. Any parameters of $f$ that are not optimized in this process are known as hyperparameters.

Once the optimal parameters $\mathbf{a}$ have been found, predictions for the targets can be made using:

$$\mathbf{y}_\mathrm{predicted} = f(\mathbf{x}_\mathrm{measured}) \tag{4}$$

Different machine learning models allow $f$ to take different analytical forms. Depending on this, the model may be able to represent a narrow or a wide set of functions. This is qualitatively defined in terms of capacity: the more flexible a model is, and the more non-linearities it can represent, the larger is its capacity. Typically models with low capacity tend to underfit the data, while models with large capacity tend to overfit the data.

In the case of the linear model, each of the $n_\mathrm{t}$ targets are calculated as:

$$y_i = a_0^i + \sum_{j=1}^{n_\mathrm{f}} a_j^i x_j \tag{5}$$

where $a_j^i$ are the parameters of the model. finding the optimal value for the parameters is equivalent to the linear regression problem, which has an analytical solution.

The quadratic model is equivalent to the linear model with the difference that instead of working directly with the input variables as features, auxiliary features are created by calculating all possible products across the input variables up to second order. For example, for an input vector

$$\mathbf{x} = (x_1, x_2, x_3), \tag{6}$$

the following vector of auxiliary features would be used:

$$\mathbf{x}_\mathrm{aux} = (1, x_1, x_2, x_3, x_1^2, x_2^2, x_3^2, x_1x_2, x_1x_3, x_2x_3). \tag{7}$$

Similarly, cubic or quartic models can be defined; the higher the degree of the polynomial, the larger the capacity of the model.

The support vector regressor (SVR)[2] model attempts to find a solution for each of the $n_t$ targets calculated as:

$$y_i = a_0^i + \sum_{j=1}^{n_e} a_j^i k(\mathbf{x}, \mathbf{x}^j) \tag{8}$$

where the summation is performed across all the examples in the training set, $a_j^i$ are parameters of the model, and $k$ is a kernel function, in this case, a Gaussian kernel defined as:

$$k(\mathbf{u}, \mathbf{v}) = \frac{1}{\sqrt{(2\pi\gamma)^{nf}}} e^{-\frac{1}{2}\gamma\|\mathbf{u}-\mathbf{v}\|^2} \tag{9}$$

where $\gamma$ is a hyperparameter of the model. While this method would in principle need to store every single example in the training set to evaluate the function, in practice, the training is performed in terms of two additional hyperparameters $C$ and $\epsilon$, finding a solution for which $a_j^i$ is 0 for most of the training examples. The examples for which $a_j^i$ is not zero are called the support vectors. For more information on the kernel functions, the training and the hyperparameters, see reference[2].

In the case of artificial neural networks (ANN)[3], the model calculates the output in a series of layers of the following kind:

$$\mathbf{x}_{l+1} = \phi(\mathbf{b}_l + \mathbf{A}_l \mathbf{x}_l) \tag{10}$$

where $\mathbf{x}_l$ is the vector with the variables at a given layer, $\mathbf{x}_{l+1}$, the values at the next layer, $\mathbf{A}_l$, a matrix of parameters of size $n_l$ x $n_{l+1}$, where $n_l$ and $n_{l+1}$ are the number of variables in the current layer and the next layer, respectively, and $\mathbf{b}_l$ is a vector of parameters with size $n_{l+1}$. The selection of the activation function $\phi$, which breaks the linearity of the model, is treated as a hyperparameter. In this case we chose the widely used rectified linear activation (ReLU) function:

$$\phi_{\mathrm{ReLU}}(x) = \begin{cases} x & \text{if} \quad x \geq 0 \\ 0 & \text{if} \quad x < 0 \end{cases} \tag{11}$$

The input to the first layer $\mathbf{x}_0$ corresponds to the input features $\mathbf{x}$, and the output of the last layer $\mathbf{x}_{N_l}$ corresponds to the targets $\mathbf{y}$, where $N_l$ is the number of layers in the ANN. The activation function is removed for the last layer so the function can output any real number. Intermediate layers calculating the internal variables $\mathbf{x}_l$ are known as hidden layers, and the number of variables used at each hidden layer is known as the number of hidden cells per layer. Both the number of hidden layers and the number of hidden cells per layer are hyperparameters of the model. By increasing these numbers the capacity of the model increases. The effect of interleaving linear multiplications with the rectified linear activation function provides an output that is linear on the inputs almost everywhere, except for the points that are exactly at 0 for the rectified linear activations. As a result, the ANN can be seen as a sophisticated piecewise function formed of a number of linear regions that increases as the number of hidden layers and hidden cells increase[4].

Training a neural network consists of finding the set of matrices $\mathbf{A}_l$ and vectors $\mathbf{b}_l$ that solve the optimization problem for the training set. This is typically performed using the iterative gradient descent technique where the derivative of the cost function with respect to each parameter of the model is calculated and used to update the values of the parameters in the direction opposite to the derivative, decreasing the value of the cost function. In this work, we use a variation of this algorithm named AdaGrad[5] for this purpose. For more information on ANN, see reference[3].

**Supplementary Note 2. Details about the variables used for the prediction.**

**Fast shot-to-shot variables.** We list here all of the fast shot-to-shot variable names used as features for prediction, currently measured at 120 Hz at the Linac Coherent Light Source (LCLS):

- *ebeamCharge* and *ebeamDumpCharge*: Electron beam charge measured at the accelerators, and at the electron dump.
- *ebeamEnergyBC1* and *ebeamEnergyBC2*: Electron beam energy measured at each of the two bunch compressors.
- *ebeamPkCurrBC1* and *ebeamPkCurrBC2*: Electron beam peak current measured at each of the two bunch compressors.
- *ebeamL3Energy*: Electron beam energy measured after the third linear acceleration stage.
- *ebeamLTUPosX* and *ebeamLTUPosY*: Horizontal and vertical electron beam positions at the Linac to Undulator (LTU) transport line.
- *ebeamLTUAngX* and *ebeamLTUAngY*: Horizontal and vertical electron beam angles at the Linac to Undulator (LTU) transport line.
- *ebeamLTU250* and *ebeamLTU450*: Electron beam position in two dispersive regions at the LTU transport line.
- *ebeamUndPosX* and *ebeamUndPosY*: Horizontal and vertical electron beam positions at the undulator.
- *ebeamUndAngX* and *ebeamUndAngY*: Horizontal and vertical electron beam angles at the undulator.
- *f_11_ENRC* and *f_12_ENRC*: Redundant X-ray total energy measurements before attenuation from two gas detectors.
- *f_21_ENRC* and *f_22_ENRC*: Redundant X-ray total energy measurements after attenuation from two gas detectors.
- *f_63_ENRC* and *f_64_ENRC*: Redundant X-ray total energy measurements corrected to be accurate for small signals (<0.5 mJ).

**Slow EPICS variables.** We list here typical slow environmental properties recorded as Experimental Physics and Industrial Control System (EPICS)[6] variables measured at 2 Hz at LCLS:

- Positions of translation stages involved in the control feedback loops.
- Voltages of power supplies involved in the control feedback loops.
- Strength of magnetic fields in the magnetic chicanes, and bending magnets.
- Nominal values for the amplitude and phases of the radiofrequency fields.
- Pressures from the vacuum systems.
- Temperatures at different stages.
- Calibration values inputted manually by operators.
- Status of beam blockers.

| | Support vector regressor | | | Artificial neural network | | | |
|---|---|---|---|---|---|---|---|
| | $C$ | $\epsilon$ | $\gamma$ | Number of hidden layers | Number of hidden cells per layer | Batch size | Training steps |
| Single-pulse photon energy | 100 | 0.08 | 0.005 | 2 | [10,5] | 1000 | 5000 |
| Single-pulse spectrum | 100 | 0.3 | 0.005 | 3 | [50,50,20] | 1000 | 5000 |
| Double-pulse delay | 100 | 0.4 | 0.005 | 2 | [50,10] | 1000 | 2400 |
| Double-pulse photon energy | 100 | 0.5 | 0.005 | 2 | [20,5] | 1000 | 2000 |

**Supplementary Table 1. Hyperparameters of the models.**

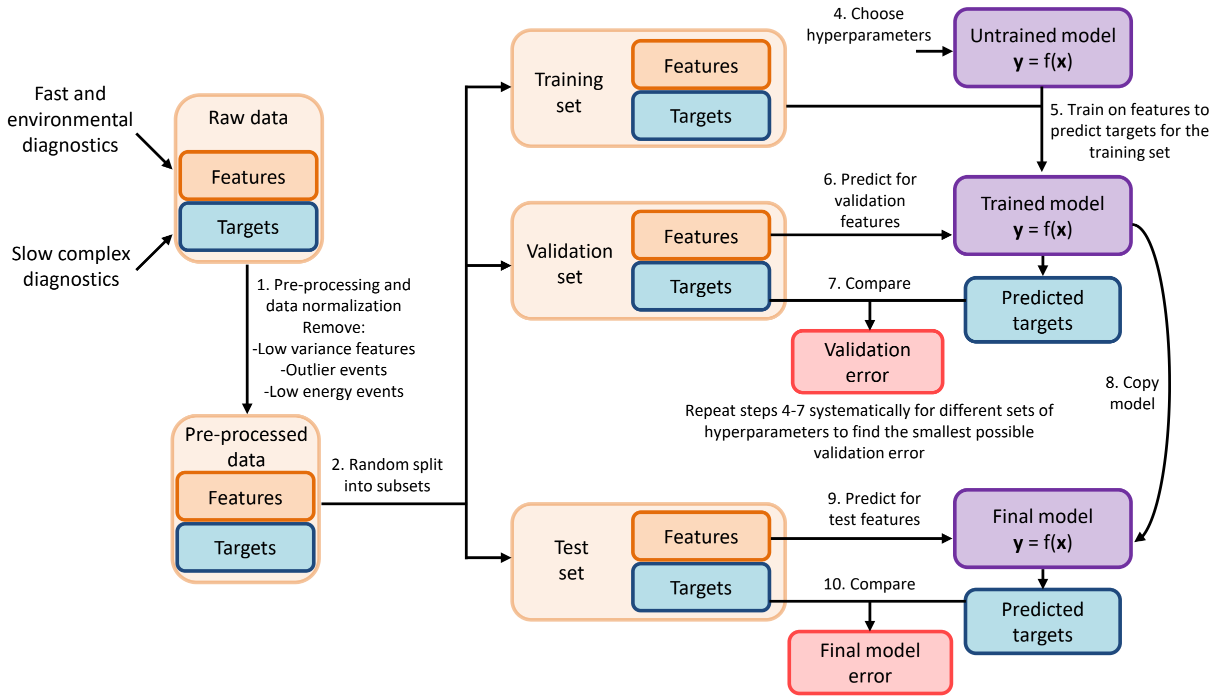


**Supplementary Figure 1. Flow diagram of the training and testing process.** After pre-processing and normalizing the input dataset, it is divided into three groups: the training set, the validation set, and the test set. Different models with different sets of hyperparameters are trained on the training set, and used to predict the targets for the validation set, allowing to obtain the validation error. Once the set of hyperparameters that yield the smallest validation error is found, the final error of the model is obtained by making predictions on the test set, which was kept isolated during the previous stages. Datasets are shown in light brown. Features are shown in orange. Targets are shown in blue. Models are shown in purple. Calculated errors in the predictions are shown in red.

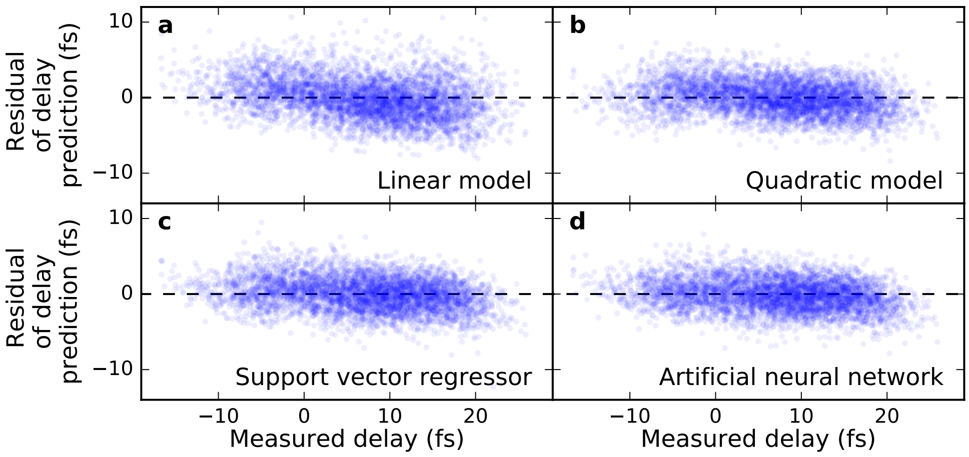


**Supplementary Figure 2. Residuals of the models predicting the time delay.** Experimental points are shown in blue. The lines showing the perfect absence of residuals are included for reference as black dashed lines. The residuals of the predictions present clear non-linear correlations with respect to the time delay for the linear and quadratic models. These non-linear correlations are greatly reduced for the support vector regressor and practically disappear for the artificial neural network.

## Supplementary References


[1]Murphy, K. P. *Machine learning: a probabilistic perspective* (MIT press, 2012).

[2]Smola, A. J. & Schölkopf, B. A tutorial on support vector regression. *Stat. Comput.* **14**, 199–222 (2004).

[3]Cheng, B. & Titterington, D. M. Neural networks: a review from a statistical perspective. *Stat. Sci.* **9**, 2–30 (1994).

[4]Montufar, G. F., Pascanu, R., Cho, K. & Bengio, Y. On the number of linear regions of deep neural networks. In *Advances in Neural Information Processing Systems*, 2924–2932 (2014).

[5]Duchi, J., Hazan, E. & Singer, Y. Adaptive subgradient methods for online learning and stochastic optimization. *J. Mach. Learn. Res.* **12**, 2121–2159 (2011).

[6]Dalesio, L. R. *et al.* The experimental physics and industrial control system architecture: past, present, and future. *Nucl. Instr. Meth. Phys. Res. A* **352**, 179–184 (1994).
